# Fortnightly or fractionated weekly docetaxel–cisplatin–5‐FU as first‐line treatment in advanced gastric and gastroesophageal junction adenocarcinoma: The randomized phase II DoGE study

**DOI:** 10.1002/cam4.3976

**Published:** 2021-05-31

**Authors:** Amélie Deleporte, Marc Van den Eynde, Frédéric Forget, Stéphane Holbrechts, Thierry Delaunoit, Ghislain Houbiers, Hassan R. Kalantari, Stéphanie Laurent, Erik Vanderstraeten, Marc De Man, Philippe Vergauwe, Marylene Clausse, Jacques Van Der Auwera, Lionel D’Hondt, Pascal Pierre, Bjorn Ghillemijn, Angelique Covas, Marianne Paesmans, Lieveke Ameye, Ahmad Awada, Francesco Sclafani, Alain Hendlisz

**Affiliations:** ^1^ Department of Medicine Gastrointestinal Unit Institut Jules Bordet Université Libre de Bruxelles Bruxelles Belgium; ^2^ Department of Medical Oncology Cliniques Universitaires Saint‐Luc Woluwe‐St‐Lambert Belgium; ^3^ Centre Hospitalier de l'Ardenne (CHA) Department of Medical Oncology Hôpital de Libramont Libramont‐Chevigny Belgium; ^4^ Department of Medical Oncology Hôpital Ambroise Paré Mons Belgium; ^5^ Department of Medical Oncology Hôpital de Jolimont La Louvière Belgium; ^6^ Department of Gastroenterology Clinique Saint Joseph Liège Belgium; ^7^ Department of Onco‐Hematology Centre Hospitalier Pelzer‐La Tourelle (CHPLT) Verviers Belgium; ^8^ Department of Gastroenterology Oncology Unit Universiteit Gent Gent Belgium; ^9^ Department of Medical Oncology AZ Maria Middelares Gent Belgium; ^10^ Department of Gastroenterology Olv Ziekenhuis Campus Aalst Aalst Belgium; ^11^ Department of Gastroenterology AZ Groeninge –Kortrijk Kortrijk Belgium; ^12^ Department of Medicine Oncology Unit Clinique Saint‐Luc Bouge Namur Belgium; ^13^ Department of Gastroenterology AZ Monica Deurne Deurne Belgium; ^14^ Department of Medical Oncology CHU UCL Namur – Site Godinne Yvoir Belgium; ^15^ Department of Medical Oncology Hôpital d’Arlon Arlon Belgium; ^16^ Department of Medicine AZ Glorieux Ronse Belgium; ^17^ Department of Statistics Institut Jules Bordet Université Libre de Bruxelles Bruxelles Belgium; ^18^ Department of Medical Oncology Institut Jules Bordet Université Libre de Bruxelles Bruxelles Belgium

**Keywords:** 5‐FU, cisplatin, docetaxel, fortnightly, gastric cancer, gastroesophageal cancer, hematological growth factors, weekly

## Abstract

**Background:**

While docetaxel/cisplatin/5‐fluorouracil (DCF) outperforms CF in first‐line gastric adenocarcinoma, toxicity remains an issue.

**Methods:**

This multicenter phase II trial randomized chemonaïve metastatic gastric adenocarcinoma patients to fractionated weekly DCF (D 40 mg/m^2^, C 35 mg/m², F 1800 mg/m² over 24 h, on days 1 and 8 every 3 weeks, arm (1) or fortnightly DCF (D 50 mg/m^2^, C 50 mg/m², F 2000 mg/m² over 48 h every 2 weeks, arm (2). Prophylactic granulocyte colony‐stimulating factor (G‐CSF) was not allowed. The primary endpoint was the rate of febrile neutropenia within the first six treatment weeks (early FN).

**Results:**

A total of 106 eligible patients were recruited. The early and overall FN rates were 9.5% and 17% in arm 1, respectively, and 5.9% and 8% in arm 2, respectively. Grade ≥3 toxicities occurred in 81% of patients in arm 1 and 90% of patients in arm 2, the most common being neutropenia (33% vs. 61%), fatigue (27% vs. 25%), vomiting (21% vs. 12%), anorexia (19% vs. 18%), and diarrhea (17% vs. 10%). Median progression‐free survival and overall survival were 5.1 (95% CI, 3.2–6.5) and 8.2 months (95% CI, 6.0–14.5), respectively, in arm 1 and 5.2 (95% CI, 3.0–6.9) and 11.9 months (95% CI, 7.4–15.9), respectively, in arm 2.

**Conclusions:**

Fractionated weekly and fortnightly DCF regimens are associated with a low risk of early FN, and a better hematological toxicity profile as compared to historical DCF without compromising efficacy. Both regimens offer greater convenience removing the need for systematic use of prophylactic G‐CSF.

## Abstract 2

The DoGE multicenter study randomised prospectively patients with chemonaïve gastric cancer between fractionated weekly and fortnightly DCF regimens. Both regimens are associated with a low risk of early FN, and a better hematological toxicity profile as compared to historical DCF without compromising efficacy. Both regimens offer greater convenience removing the need for systematic use of prophylactic G‐CSF.

## 1 INTRODUCTION

Gastric cancer (GC) is the third leading cause of cancer‐related death worldwide.^1^ Approximately one third of patients present with metastatic disease at diagnosis and the majority of those who undergo curative‐intent treatment including surgery and peri‐operative chemotherapy for localized disease experience tumor recurrence.^2^ While the therapeutic armamentarium for this disease has enriched over time with agents such as anti‐HER‐2, VEGFR2 monoclonal antibodies, and immune checkpoint inhibitors,^3^, ^4^, ^5^, ^6^, ^7^, ^8^, ^9^ cytotoxic chemotherapy remains the mainstay of treatment both in the early stage and metastatic setting.^10^

Cisplatin (C) combined with 5‐fluorouracil (F) has long been a standard first‐line treatment for gastric and gastroesophageal junction (GEJ) adenocarcinoma, being historically associated with an objective response rate of approximately 20% and a median overall survival (mOS) of 7.2 months.^11^ Docetaxel (D) has shown activity in GC without evidence of cross‐resistance with platinum compounds.^12^, ^13^, ^14^ When combined with C and F (DCF) in a 3‐weekly regimen, it increases response rate (RR), median time to progression (mTTP), and mOS as compared to CF, but at the cost of higher rates of grade ≥3 adverse events (69% vs. 59%) and febrile neutropenia (FN) (29% vs. 12%).^15^ Therefore, routine use of DCF in clinical practice for patients with advanced gastric and GEJ adenocarcinoma remains limited.

Over the last two decades, efforts have been made to develop docetaxel‐based triplet chemotherapy regimens which could be as effective as, but less toxic than the conventional DCF regimen. Generally, these consisted of studies which investigated dose modifications of the 3‐weekly DCF schema or alternative, either weekly or 2‐weekly, split dosing regimens. Overall, antitumor activity and efficacy were maintained, and a substantial improvement of the safety profile was also observed especially with regards to the risk of hematological toxicity.^16^ Additionally, modified DCF regimens provide some practical advantages such as, for instance, no need for any pre‐ and/or post‐treatment hydration and inpatient admission when cisplatin is given at a dose of ≤50 mg/m². Nevertheless, there is no universal consensus regarding the most convenient modified DCF regimen to use in routine care or clinical research, and practices vary substantially from one institution to another.

Here, we report the results of the Docetaxel in Gastric cancer treatment Evaluation (DoGE) study, a randomized phase II trial testing the safety and efficacy profile of two novel modified DFC regimens in the first‐line setting of advanced gastric and GEJ adenocarcinomas.

## 2 MATERIALS AND METHODS

### 2.1 *Patient* *eligibility*

Patients had to be diagnosed with a previously untreated, histologically confirmed, RECIST (version 1.0)‐assessable, advanced, or metastatic gastric or GEJ adenocarcinoma. Other key inclusion criteria included age ≥18 years, adequate organ function, and an Eastern Cooperative Oncology Group (ECOG) performance status of <2. Prior systemic chemotherapy or chemoradiotherapy (without taxanes) for locoregional disease was allowed if completed more than 6 months prior to study inclusion. If cisplatin was administered as part of this treatment, the total administered dose had to be <400 mg/m^2^.

The study (EudraCT 2008‐000551‐10) was approved by a central ethics committee, local ethics committee from each participating center, and the relevant Belgian authorities. The trial was conducted according to the International Conference on Harmonization Good Clinical Practice guidelines and the declaration of Helsinki. All patients signed an informed consent to confirm their willingness to participate in the study before any study procedure was performed.

### 2.2 *Study* *design*

**FIGURE 1:**
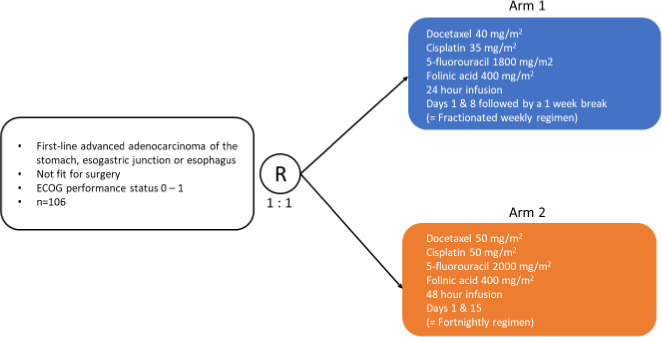
DoGE study design

This was a multicenter, open‐label, randomized phase II trial. Eligible patients were randomly allocated (1:1 ratio) to one of two treatment arms (Figure 1). In arm 1 (weekly regimen) patients received treatment for two consecutive weeks (on days 1 and 8) every 3 weeks. Treatment consisted of D (40 mg/m^2^ over 60 min), C (35 mg/m² over 30 min), FA (400 mg/m² or 200 mg/m² in the levogyre form over 60 min), and F (1800 mg/m² over 24 h). A 500 ml of saline solution (NaCl 0.9%) was administered during the infusion of D, C, and FA. In arm 2 (fortnightly regimen) patients received treatment once every 2 weeks. Treatment consisted of D (50 mg/m^2^ over 60 min), C (50 mg/m² over 30 min), FA (400 mg/m² or 200 mg/m² in the levogyre form over 60 min), and F (2000 mg/m² over 48 h). A one liter saline solution was administered before and after the infusion of C.

In both arms, corticosteroid premedication was mandatory with 8 mg of dexamethasone administered intravenously prior to the infusion of D, followed by 8 mg of dexamethasone per day given orally for 3 days. A bolus injection of furosemide 20 mg was mandatory before the infusion of C. Treatment was continued until the development of toxicities which were considered unacceptable either by the patient (i.e., jeopardizing their mental or physical wellbeing/quality of life) or by the treating physician (i.e., jeopardizing patient safety), patient's refusal to continue or disease progression, whichever occurred first. Use of hematopoietic growth factors, including granulocyte colony‐stimulating factors (G‐CSF), was only allowed as secondary prevention and based on the discretion of the treating physician.

Toxicities were graded according to the National Cancer Institute Common Toxicity Criteria for Adverse Events (CTCAE, version 3.0). In the event of myelotoxicity, treatment was delayed until recovery (i.e., ANC ≥ 1500/mm³ and PLT ≥ 100 000/mm³ for day 1 in either arm, and ANC ≥ 1000/mm³ and PLT ≥ 75 000/mm³ for day 8 in arm 1). No maximum delay was defined, but after 2 weeks the decision to discontinue treatment was left to the investigator's discretion.

Response to treatment was assessed every 6 weeks in both arms according to the RECIST v1.1 criteria.

### 2.3 Statistical considerations

The main objective of the study was to evaluate the acute hematological toxicity of both regimens. The primary endpoint was the rate of FN within the first 6 weeks of treatment (i.e., within the first two cycles in arm 1, and within the first three cycles in arm 2). In order to exclude an insufficiently active regimen, the rate of disease control after 6 weeks of treatment was also assessed. In each arm, a Bryant and Day design was used allowing early study discontinuation for either futility or excessive toxicity according to the following assumptions: for toxicity, a rate of FN of <10% would qualify the regimen as acceptable, while a rate of >25% would be considered as unacceptable. The probability of accepting a false positive result (i.e., considering sufficiently safe a regimen with a high rate of FN) was set at 15%. For efficacy, a disease control rate of <70% was considered unacceptable while a disease control rate of >85% was considered worthy of further investigation. The probability of accepting a false positive result (i.e., considering sufficiently active a regimen with insufficient activity) was set at 10%. Overall, a regimen with activity and a favorable toxicity profile was to be considered of interest with a probability of 90%. Based on these assumptions, 28 evaluable patients were required for initial assessment in each arm. If >7 patients experienced at least one episode of FN, or <21 patients had disease control after 6 weeks of study treatment, the corresponding arm would be closed prematurely. Otherwise, accrual would continue until 63 evaluable patients were recruited in each arm. After full study recruitment, treatment would be considered of interest if <13 patients experienced at least one episode of FN and ≥49 patients had disease control after 6 weeks of treatment.

No formal comparison was planned between the study arms. Randomization was performed at the Institut Jules Bordet data center using the minimization technique and performance status (ECOG PS 0 or 1) as stratification factor.

Confidence intervals (CIs) for binary variables were calculated using the Wilson method. Progression‐free survival (PFS) was defined as the time from randomization to progression or death, whichever occurred first. Overall survival (OS) was defined as the time from randomization to death regardless of cause. Patients who were alive without progression at the last follow‐up were censored. Time to significant event (TTSE) was defined as the time from randomization to grade >2 toxicity, progression or death, whichever occurred first. TTSE, PFS, and OS were assessed with Kaplan–Meier curves. Median survival was calculated with 95% CIs. Relative dose intensity of each chemotherapy agent was compared between the two arms using Wilcoxon test.

## 3 RESULTS

### 3.1 *Study* *population*

Between October 2008 and October 2013, 106 eligible patients (53 in each arm) were recruited across 15 Belgian centers. The study was closed prematurely due to poor accrual. Patient demographics and baseline characteristics are summarized in Table 1. Treatment arms were overall well balanced for most variables. Median age was 61 in arm 1 and 64 in arm 2. The majority of patients in either arm had metastatic disease while only one third had undergone prior surgery for the primary tumor. Adjuvant chemotherapy was administered in 12% and 11% of cases in arm 1 and arm 2, respectively.

**TABLE 1:** Patients characteristics

	Arm 1 Fractionated weekly regimen (*N* = 53)	Arm 2 Fortnightly regimen (*N* = 53)
Age at randomization		
Mean ± SD	60 ± 10	63 ± 10
Median (min–max)	61 (33–85)	64 (40–83)
Gender		
Male	37 (70%)	39 (74%)
Female	16 (30%)	14 (26%)
Histology		
Intestinal type adenocarcinoma	20 (38%)	16 (30%)
Diffuse type adenocarcinoma	18 (34%)	24 (45%)
Adenocarcinoma (no other specification)	12 (23%)	13 (25%)
Other	2 (4%)	0
Missing info	1 (2%)	0
Differentiation		
Well	2 (4%)	7 (13%)
Moderately	21 (40%)	15 (28%)
Poor	23 (44%)	20 (38%)
Unknown	6 (12%)	11 (21%)
Missing info	1	
Stage at study entry		
IIIA	3 (6%)	5 (10%)
IIIB	2 (4%)	4 (8%)
IV	42 (89%)	42 (82%)
Missing info	6	2
Prior surgery for gastric or GEJ cancer?		
No	35 (67%)	35 (66%)
Yes	17 (33%)	18 (34%)
Missing info	1	
If yes, type of surgery		
Total gastrectomy	7	8
Partial gastrectomy	2	2
Other	8	8
Adjuvant chemotherapy for completely resected early stage gastric or GEJ cancer		
No	46 (88%)	47 (89%)
Yes	6 (12%)	6 (11%)
Missing info	1	

Among the 106 patients included, 103 received the assigned treatment (52 in arm 1 and 51 in arm 2), one patient withdrew consent 5 days after inclusion, one developed an intestinal obstruction, and one died of progressive disease on the 13th day after inclusion without receiving any treatment.

### 3.2 Treatment compliance

The median number of cycles of chemotherapy was 4 (range 1–19) in arm 1 and 7 (range 1–30) in arm 2 (Table 2). Among patients who received at least 2 cycles, 96% (43/45) of those in arm 1 and 83% (40/48) of those in arm 2 had at least one dose reduction or treatment delay throughout the study treatment. Toxicity was the cause of treatment discontinuation in 40% (21/52) and 35% (18/51) of cases, respectively, in arm 1 and arm 2. Treatment was completed as per study protocol in 37% (19/52) of patients in arm 1 and in 39% (20/51) of patients in arm 2, respectively. Relative dose intensities of D, C, and F were similar between the two arms (*p* = 0.94, 0.93, and 0.79, respectively) (Table 3).

**TABLE 2:** Number of cycles and prevalence of treatment delays/dose reductions

	Arm 1 Fractionated weekly (*N* = 52)	Arm 2 Fortnightly (*N* = 51)
Number of cycles		
Median (range)	4 (1–19)	7 (1–30)
1	7 (13%)	3 (6%)
≥2	45 (87%)	48 (94%)
No dose reduction or delay^a^	2	8
At least one dose reduction or delay^a^	43	40

^a^ The notion of treatment delay/dose reduction is only applicable in patients with at least two cycles.

**TABLE 3:** Chemotherapy dose intensities

Drug: % median (interquartile range)	Arm 1 Fractionated weekly (*N* = 52)	Arm 2 Fortnightly (*N* = 51)
Docetaxel (D)	73 (62–89)	73 (64–89)
Cisplatin (C)	72 (59–88)	71 (61–88)
5‐FU (F)	73 (61–91)	73 (64–88)

### 3.3 Toxicity

Less than 10% of patients in either arm experienced FN within the first 6 weeks of treatment: 9.5% (5/52) in arm 1 and 5.9% (3/51) in arm 2. Of these, three died of sepsis within 2 weeks after the onset of FN. Throughout the treatment duration, 17% of patients in arm 1 and 8% in arm 2 experienced FN.

Table 4 reports grade ≥3 toxicities. These were reported in 81% of patients in arm 1 and 90% in arm 2, and consisted mainly of neutropenia (33% vs. 61%), fatigue (27% vs. 25%), vomiting (21% vs. 12%), anorexia (19% vs. 18%), and diarrhea (17% vs. 10%).

**TABLE 4:** Grade ≥3 toxicities

	Arm 1 Fractionated weekly (*N* = 52)		Arm 2 Fortnightly (*N* = 51)	
	*N* (%)	(95% CI)	*N* (%)	(95% CI)
AE grade ≥3	42 (81)	(68–89)	46 (90)	(79%–96%)
Hematological AE grade ≥3	22 (42)	(30–56)	33 (65)	(51%–76%)
Anemia	7 (13)	(7–25)	7 (14)	(7%–26%)
Neutropenia	17 (33)	(22–46)	31 (61)	(47%–73%)
Febrile neutropenia	9 (17)	(9–30)	4 (8)	(3%–19%)
Thrombocytopenia	6 (12)	(5%–23%)	3 (6)	(2%–16%)
Thromboembolic event	2 (4)	(1%–13%)	1 (2)	(0.4%–10%)
Non hematogical AE grade ≥3	32 (62)	(48%–74%)	24 (47)	(34%–60%)
Alteration of liver function test	6 (12)	(5%–23%)	1 (2)	(0.4%–10%)
Total protein	–		–	
Alteration of renal function	–		–	
Fatigue	14 (27)	(17%–40%)	13 (25)	(16%–39%)
Anorexia	10 (19)	(11%–32%)	9 (18)	(10%–30%)
Weight loss	1 (2)	(0.3%–10%)	2 (4)	(1%–13%)
Nausea	7 (13)	(7%–25%)	2 (4)	(1%–13%)
Vomiting	11 (21)	(12%–34%)	6 (12)	(6%–23%)
Diarrhea	9 (17)	(9%–30%)	5 (10)	(4%–21%)
Constipation	–		–	
Stomatitis	1 (2)	(0.3%–10%)	4 (8)	
Allergic reaction	–		1 (2)	(0.4%–10%)
Motor neuropathy	2 (4)	(1%–13%)	1 (2)	(0.4%–10%)
Sensory neuropathy	3 (6)	(2%–16%)	1 (2)	(0.4%–10%)
Alopecia	2 (4)	(1%–13%)	2 (4)	(1%–13%)

Abbreviation: AE, adverse events.

### 3.4 Efficacy

**FIGURE 2:**
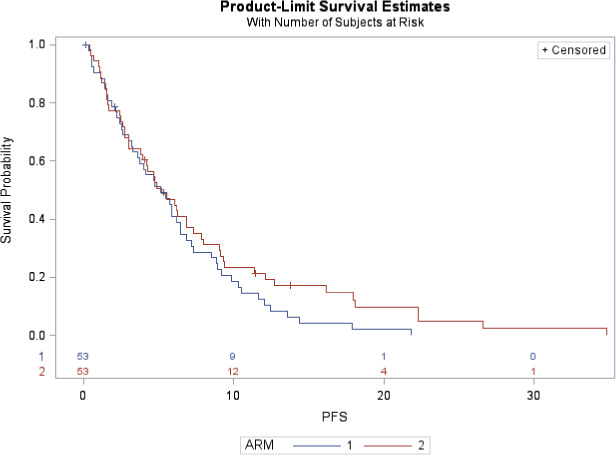
Progression‐free survival

**FIGURE 3:**
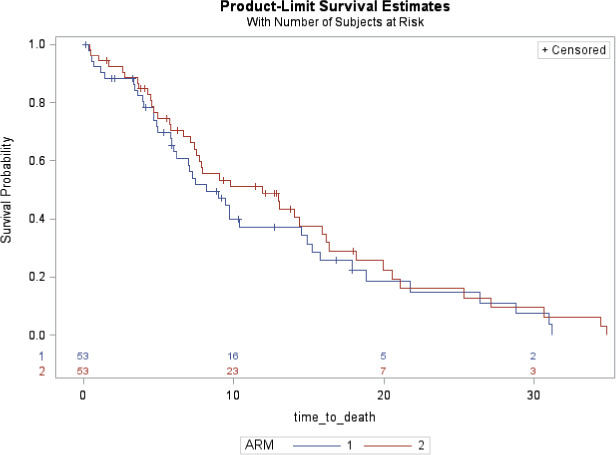
Overall survival

Forty‐five patients in each arm received at least 6 weeks of treatment. The overall response rate (ORR; i.e., complete plus partial responses) was 49% in arm 1 and 44% in arm 2 (Table 5). The 6‐week disease control rate (DCR) was 83% (95% CI: 71–91) in arm 1 and 79% (95% CI: 67–88) in arm 2. In the same arms, median TTSE was 2 weeks (95% CI: 1–6) and 3 weeks (95% CI: 2–5), median mPFS was 5.1 months (95% CI: 3.2–6.5) and 5.2 months (95% CI: 3.0–6.9), and mOS was 8.2 months (95% CI: 6.0–14.5) and 11.9 months (95% CI: 7.4–15.9), respectively (Figure 3). At 6 months the OS rate was 63.1% (±7.0%) in arm 1 and 70.5% (±6.4%) in arm 2 (Figure 2).

**TABLE 5:** Best response

	Arm 1 Fractionated weekly At least two chemo cycles (*N* = 45)	Arm 2 Fortnightly At least three chemo cycles (*N* = 45)
	*N* (%)	*N* (%)
Complete response	2 (4)	2 (4)
Partial response	20 (44)	18 (40)
Stable disease	16 (36)	16 (36)
Progression	6 (13)	4 (9)
Not evaluable	1 (2)	5 (11)

## 4 DISCUSSION

This study confirms that, in a population of chemotherapy‐naïve, advanced gastric or gastroesophageal junction adenocarcinoma patients treated with a triplet DCF‐containing therapy, fractionated schedules are associated with reduced rates of FN compared to the historical DCF regimen, while maintaining satisfactory oncological outcomes.

Most of the recent advances in the management of advanced GC have mostly been secondary to the increased number of active treatment options in the refractory setting. With the only exception of the addition of the anti‐HER2 monoclonal antibody herceptin or, more recently, the immune checkpoint inhibitor nivolumab to standard first‐line doublet chemotherapy,^3^, ^8^, ^9^ virtually no outcome improvement has been achieved through the intensification of first‐line cytotoxic chemotherapy, and platinum‐based doublet regimens are still regarded as the preferred choice in this setting.

This is in contrast with other gastrointestinal tumors such as pancreatic and colorectal cancer, where triplet chemotherapy regimens have been demonstrated to improve the oncological outcomes, and are now endorsed by international guidelines and commonly used in clinical practice.^17^, ^18^

The main reason behind this discrepancy is the poor safety profile of the intensified regimens which have historically been tested in GC. DCF has been the prototype of triplet chemotherapy in this setting. In the pivotal V‐352 trial, combined administration of D (75 mg/m^2^, day 1), C (75 mg/m^2^, day 1), and F (750 mg/m^2^/day, days 1–5) for every 3 weeks was shown to significantly reduce the risk of progression and death by 32% and 23%, respectively, compared to standard CF.^15^ Furthermore, it significantly prolonged the time to definitive worsening of performance status and deterioration of global health status/QOL. Nevertheless, this regimen was associated with higher rates of treatment delays (64% vs. 42%), grade ≥3 treatment‐related toxicities (69% vs. 59%), grade ≥3 neutropenia (82% vs. 57%), and complicated neutropenia (29% vs. 12%). As a result, the interest for the outcome data potentially achievable with this intensified chemotherapy treatment was promptly tempered by its unsatisfactory safety profile. The rates of grade ≥3 and complicated neutropenia were particularly concerning, and these did not appear to improve substantially when granulocyte colony‐stimulating factor was regularly used.^19^

Since then, numerous studies have been conducted to explore alternative, less toxic DCF regimens. In a randomized phase III trial from China, a modified DCF regimen (D and C 60 mg/m^2^ each plus F 600 mg/m^2^/day, days 1–5, every 3 weeks) was confirmed to be superior to standard CF in terms of ORR, PFS, and OS. Nevertheless, toxicity of the experimental treatment remained an issue with higher rates of grade ≥3 treatment‐related adverse events (77.3% vs. 46.1%), grade ≥3 neutropenia (60.5% vs. 9.6%), and complicated neutropenia (14% vs. 0%) compared to the control arm.^20^ Moreover, a meta‐analysis of 24 studies and 1311 patients who were treated with weekly, biweekly, or reduced dose 3‐weekly schedules of DCF, showed a manageable safety profile with grade ≥3 neutropenia and FN occurring in 29.1% and 7.6% of patients, respectively. Oncological outcomes were not affected by treatment de‐intensification with an ORR of 49%, a median PFS of 7.2 months, and a median OS of 12.3 months being reported.^16^

While modified DCF regimens have been extensively investigated in esophago‐gastric cancer patients, to the best of our knowledge, the two DCF schemes used in this trial have never been tested. In both treatment arms, we succeed to keep the rate of early FN (i.e., within 6 weeks after treatment start) below 10% in accordance with our statistical hypothesis, without the prophylactic use of G‐CSF.

In the weekly arm, 9.5% of patients experienced early FN while this occurred in only 5.9% of patients who were allocated to the fortnightly arm. Although the rate of early FN in the V‐352 was not reported and no direct comparison is therefore possible with the findings from this study, it is worth noting that the overall rate of FN in our population (17% for the weekly arm and 8% for the fortnightly arm) appeared lower than that observed in the V‐352 trial (29%).^15^ Interestingly, the planned dose intensity in our study was not substantially different compared to that of the V‐352 trial. In the latter and in our weekly and fortnightly arms, respectively, this was 25, 26.7, and 25 mg/m^2^/week for D, 25, 23.3, and 25 mg/m^2^/week for C, and 1250, 1200, and 1000 mg/m^2^/week for F.^15^ Bearing in mind the limitations of inter‐trial comparisons, these data suggest that the lower risk of FN observed in our study is likely secondary to simple dose fractionation.

While FN was our primary safety measure, important insights can be gained also from the analysis of the overall study safety profile. This appeared slightly different between the two arms with a higher proportion of patients in the weekly arm experiencing non‐hematological toxicities (62% vs. 47%). In particular, a higher risk of gastrointestinal adverse events (51% vs. 26%) including nausea, vomiting, and diarrhea was reported for patients treated with weekly chemotherapy. This is in line with the 49% reported by the investigators of the V‐352 trial.^15^ Also, and in contrast with the observed FN rates, treatment in the fortnightly arm was associated with a higher risk of grade ≥3 neutropenia (61% vs. 33%). It should be noted, however, that our trial was not designed to allow a formal comparison between the two arms and any difference in terms of safety should be interpreted with caution. It is reassuring, however, that dose/schedule modifications in either arm did not appear to affect the oncological outcomes. The ORRs and survival outcomes were similar between the two treatment arms and in line with those previously reported in trials of classical or modified DCF regimens in the same setting.^16^

Our study has some limitations. First of all, trial accrual was discontinued when only 84% of the planned recruitment had been reached ultimately affecting our ability to formally assess the pre‐defined statistical hypothesis. Furthermore, the lack of a proper control group of patients treated with either standard or modified DCF do not allow us to draw any definitive conclusion regarding safety and efficacy of the regimens investigated. As a result, our study should be regarded as exploratory in nature and any interpretation of the results especially when compared against historical reports should be carefully weighed. Finally, it should be noted that the vast majority of our study patients still suffered grade ≥3 adverse events (81% in the weekly arm and 90% in the fortnightly arm). These data highlight that, despite the relatively low rate of FN here reported, there is still scope for the improvement of the safety profile of modified DCF regimens, and patient selection remains of paramount importance when considering treatment intensification with first‐line triplet chemotherapy. Despite these limitations, however, our study has the merit to investigate safety and efficacy of two new modified DCF regimens, thus adding substantially to the available evidence on treatment de‐intensification in the first‐line setting of advanced esophago‐gastric cancer.

Since our study was conducted, important advances have been made in the management of GC, with active and manageable triplet chemotherapy regimens with or without taxanes being implemented in routine practice or evaluated in clinical trials. FLOT is now a standard of care peri‐operative treatment for gastric and gastroesophageal junction adenocarcinoma, and is increasingly used in patients with advanced disease.^2^, ^21^ Promising efficacy data have also been reported with the repurposing of FOLFIRINOX in the metastatic setting of gastroesophageal cancer.^22^ As a result, the interest for modified DCF regimens has gradually reduced, this being further mitigated by the negative results of recent trials comparing DCF‐like regimens with standard doublet chemotherapy.^23^

In conclusion, we showed that dose fractionation of DCF within a weekly or fortnightly scheme may be a valuable option to reduce the risk of FN which is generally associated with the standard DCF regimen. This can be achieved without the need for systematic use of prophylactic G‐CSFs and, more importantly, without the drawback of suboptimal oncological outcomes.

## CONFLICT OF INTEREST

Dr Deleporte and Ghillemijn report grants from Sanofi during the conduct of the study. Dr Awada reports advisory roles, travel grants, and speaker fees from Roche, Lilly, Amgen, ESAI, BMS, Pfizer, Novartis, MSD, Ipsen, and Leopharma. Dr Hendlisz reports research grants from Pfizer, Sanofi‐Aventis, and Sirtex.

## Supporting information

Fig S1‐S3
