# Supplementary material for: Fortnightly or fractionated weekly docetaxel–cisplatin–5‐FU as first‐line treatment in advanced gastric and gastroesophageal junction adenocarcinoma: The randomized phase II DoGE study
Source: Cancer Med. 2021 May 31;10(13):4366–74. doi: 10.1002/cam4.3976 (PMC8267119; doi:10.1002/cam4.3976)
Supplement: Supplementary file 1 — Fig S1‐S3 [file CAM4-10-4366-s001.docx]

**Supplemental files**

**Supplemental Figure 1: DoGE study design**

**
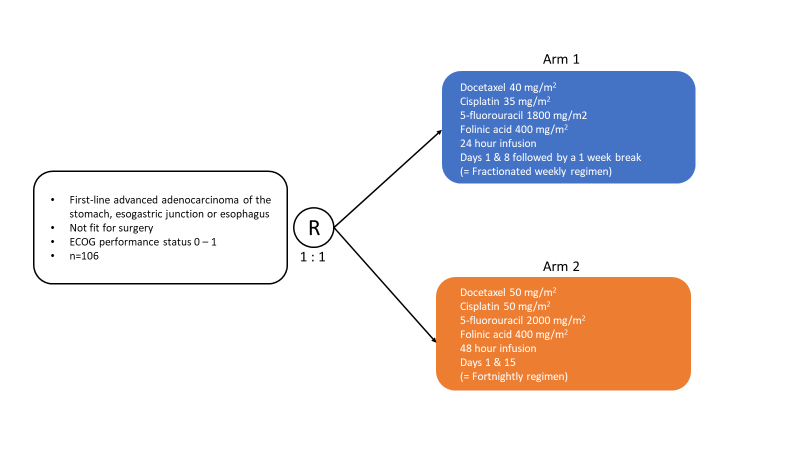
**

Supplemental figure 2: Overall survival

**
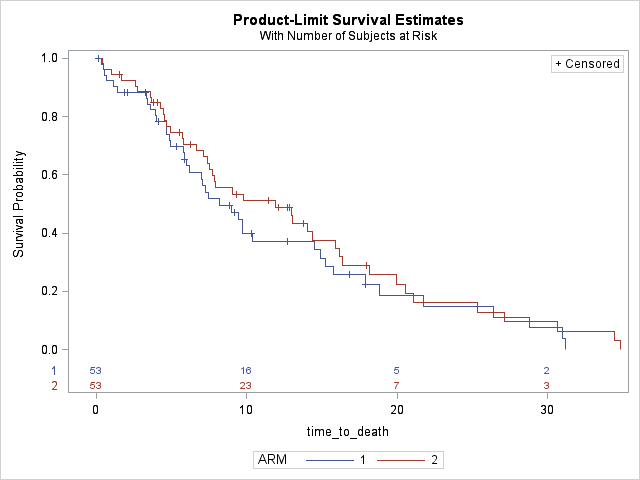
**

Supplemental figure 3: Progression-free survival

**
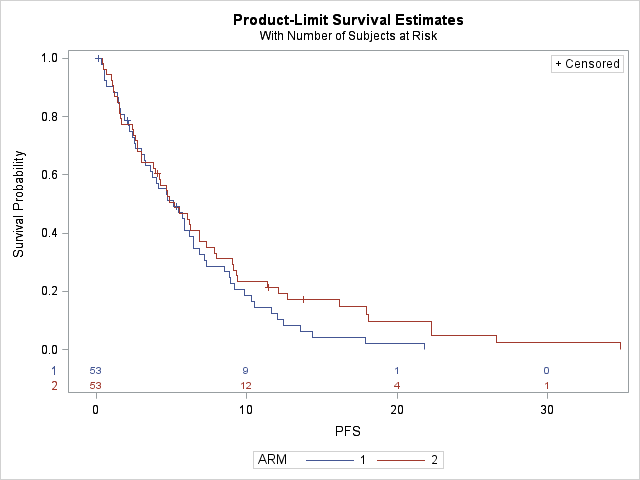
**
